# An Open-Label Study of an Herbal Topical Medication (QoolSkin) for Patients with Chronic Plaque Psoriasis

**DOI:** 10.1100/tsw.2007.111

**Published:** 2007-07-03

**Authors:** Arnon D. Cohen, Raquel Shalev, Ron Yaniv, Avner Shemer

**Affiliations:** ^1^ Clalit Health Services, Faculty of Health Sciences, Beer-Sheva, Israel; ^2^ Siaal Research Center for Family Medicine and Primary Care, Faculty of Health Sciences, Beer-Sheva, Israel; ^3^ Department of Dermatology, Sheba Medical Center, Tel Hashomer, Sackler School of Medicine, Tel Aviv University, Tel Aviv, Israel; ^4^ Maccabbee Health Services, Israel

**Keywords:** psoriasis, herbal medication, Israel

## Abstract

QoolSkin is novel herbal topical medication indicated for the treatment of patients with psoriasis and we endeavored to determine the efficacy of QoolSkin in patients with chronic plaque psoriasis. In an open-label, parallel-group study conducted at four sites in Israel, patients with chronic plaque psoriasis were treated by application of QoolSkin two to three times per day, for a period of 16 weeks. Clinical assessment was performed using the Psoriasis Area and Severity Index (PASI) and the Beer-Sheva Psoriasis Severity Score (BPSS). The study included 100 patients (48 men, 52 women; age 18–65 years). QoolSkin was well tolerated and there were no local or systemic side effects. There was a 19% reduction in PASI, from a mean of 9.8 ± 9.5 before treatment to 8.0 ± 9.6 after treatment (p = 0.09). There was a 20% reduction in BPSS, from a mean of 16.1 ± 9.8 before treatment to 12.8 ± 10.6 after treatment (p = 0.01). The reduction in PASI and BPSS was pronounced in women (32 and 31%, respectively) as compared to men (9 and 11%, respectively). The reduction in PASI and BPSS was parallel to the length of time the patients were treated by QoolSkin. In patients treated by one of the investigators, who applied QoolSkin three times per day and for a long period of time (mean 101.1 days), the reduction in PASI was 32.0% and the reduction in BPSS was 37.8%. In patients with chronic plaque psoriasis, QoolSkin treatment was well tolerated. Application of QoolSkin was associated with a decrease in disease severity, as assessed by the patients and physicians. Application of QoolSkin three times per day for long period is associated with a better response to treatment.

### INTRODUCTION

Psoriasis is a chronic disorder characterized by erythematous scaly patches that affect the scalp, trunk, extensor surfaces of the limbs, and the genital area. Psoriasis is highly prevalent in the general population, mainly as a result of its chronicity and the absence of a cure. Patients with psoriasis have high rates of depression and a decreased quality of life. Patients with mild to moderate psoriasis are usually treated with topical treatments. Systemic therapy or phototherapy are reserved for patients with moderate to severe disease[1,2,3,4].

Recent studies described the role of complementary and alternative medicine in patients with psoriasis, including mind-body interventions, herbal medicine, nutrition, and traditional Chinese medicine[5,6,7,8,9,10,11,12,13,14,15,16,17,18]. In a recent study by Ben-Arye et al.[19], it was shown that 18% of Israeli patients with psoriasis used homeopathic medicines.

QoolSkin is an herbal topical medication indicated for the treatment of patients with psoriasis. QoolSkin ingredients include riboflavin, citrus medica limonum (lemon) juice, vinegar, taraxacum oficinalis (danadelion) extract, cereus grandiflorus (cactus) flower extract and opuntia coccinellifera fruit extract, ascorbic acid, calcium ascorbate, sodium ascorbate, and tartaric acid. Uncontrolled observations have shown that QoolSkin is beneficial for patients with psoriasis as reported by patients and complementary medicine personal. However, to date, we have no data on the effect of QoolSkin on psoriasis observed by dermatologists in a planned clinical trial. Therefore, we conducted a clinical trial of QoolSkin in patients with psoriasis. The primary objective of this study was to determine the efficacy of QoolSkin topical treatment in patients with chronic plaque psoriasis.

### METHODS

This was an open-label, parallel-group study to evaluate the tolerability of QoolSkin treatment. The study was conducted at four sites in Israel. Approval for the study was obtained from the Institutional Review Board and written informed consent was obtained from each patient prior to enrollment in the study. The study was conducted in accordance with the ethical principles outlined in the Declaration of Helsinki.

#### Population

Patients 18 years of age or older with chronic plaque psoriasis were eligible to participate in this study. Nursing mothers and pregnant women were excluded, as were patients with erythrodermic, pustular, or guttate psoriasis. Exclusion criteria also included patients who had been treated with any systemic antipsoriatic systemic drug within the previous 4 weeks as well as patients with immune suppression.

#### Treatment

QoolSkin is an herbal topical medication indicated for the treatment of patients with psoriasis. The complete list of QoolSkin ingredients include water, glycerin, riboflavin, citrus medica limonum (lemon) juice, vinegar, taraxacum oficinalis (danadelion) extract, cereus grandiflorus (cactus) flower extract, opuntia coccinellifera fruit extract, ascorbic acid, calcium ascorbate, sodium ascorbate, and tartaric acid. The patients were instructed to apply QoolSkin formulation on affected skin twice a day (in patients treated by investigators RS, ADC, and RY) or three times per day (investigator AS).

#### Study Design

The study was designed as an open-label, parallel-group study conducted at four sites in Israel and included assessments at weeks 2, 4, 8, and 16 after enrollment in the study. It was possible for patient who had a good response to QoolSkin to continue the treatment for a longer time period.

### Efficacy Assessments

Clinical response was measured using the Psoriasis Area and Severity Index (PASI) and the Beer-Sheva Psoriasis Severity Score (BPSS). The PASI ranges from 0 to 72 and is based on an equation that combines the percentage of affected body surface area (BSA) with an assessment of the extent and severity (erythema, desquamation, and induration) of the psoriatic lesions at four anatomic sites (head, upper extremities, trunk, and lower extremities).

In addition to the PASI, we used the BPSS. This is a novel tool for the ambulatory assessment of patients with psoriasis that has been described by us previously[20]. In the current study, we used BPSS version 2, which has 16 items, 9 of which are recorded by the physician and 7 by the patient, using linear 5- or 6-point visual analogue scales. All scores are summed directly and BPSS ranges from 0 to 71.

An additional objective of this study was an assessment of the tolerability of QoolSkin, which was achieved by assessing local side effects at each visit.

### Statistical Analyses

Safety analyses were carried out on all patients who received QoolSkin. Analyses of efficacy measures were based on the intent-to-treat cohort. Descriptive statistics were used to summarize efficacy end-points. Results of continuous variables are shown as means ± SD. To analyze statistically significant differences in continuous parameters before and after treatment, paired t-tests were used. Dichotomous variables were analyzed using chi square tests. Logistic and linear regressions were used for multivariate analyses. $p$ values $\leq 0.05$ were considered statistically significant.

## RESULTS

The study included 100 patients (48 men, 52 women; age 18–65 years); 36% of the patients had a positive family history of psoriasis, 24% of the patients had a history of arthritis or joint pains. The mean length of treatment was 88.1 days (range: 7–253 days). QoolSkin was well tolerated and there were no local or systemic side effects.

There was a 19% reduction in PASI, from a mean of 9.8 ± 9.5 before treatment to 8.0 ± 9.6 after treatment ($p = 0.09$). There was a 20% reduction in BPSS, from a mean of 16.1 ± 9.8 before treatment to 12.8 ± 10.6 after treatment ($p = 0.\ 01$) (Table 1).

The reduction in PASI and BPSS was pronounced in women (32 and 31%, respectively) as compared to men (9 and 11%, respectively) (Table 2). The reduction in PASI and BPSS increased with the length of time QoolSkin was applied to the skin (Table 3). The response to QoolSkin treatment was different between the four study sites, according to the treating investigator (Table 4).

The effect of QoolSkin was pronounced in patients treated by one of the investigators (AS). These patients applied QoolSkin three times per day and for a long period of time (101.1 ± 43.1 days, range 14–171 days) (Table 5).

## DISCUSSION

Results of this open-label study show that QoolSkin was well tolerated. At the four study sites, no adverse events were observed. Application of QoolSkin was associated with a decrease in disease severity, as assessed by the patients and physicians.

**TABLE 1**
**Assessment of Psoriasis Severity Before and After QoolSkin Treatment**

| | Before QoolSkin Treatment | | | | After QoolSkin Treatment | | | |
|---|---|---|---|---|---|---|---|---|
| | **Min.** | **Max.** | **Mean** | **SD** | **Min.** | **Max.** | **Mean** | **SD** |
| Patients' assessment | | | | | | | | |
| General assessment | 1 | 5 | 2.8 | 1.3 | 0 | 5 | 2.2 | 1.5 |
| Pruritus | 0 | 5 | 2.4 | 1.6 | 0 | 5 | 1.6 | 1.6 |
| Face involvement | 0 | 5 | 0.5 | 1.0 | 0 | 5 | 0.5 | 1.0 |
| Nails involvement | 0 | 5 | 0.4 | 1.0 | 0 | 5 | 0.4 | 1.0 |
| Palms involvement | 0 | 5 | 0.4 | 1.1 | 0 | 4 | 0.3 | 0.8 |
| Soles involvement | 0 | 5 | 0.4 | 1.1 | 0 | 5 | 0.4 | 1.1 |
| Genital involvement | 0 | 5 | 0.7 | 1.2 | 0 | 5 | 0.5 | 1.1 |
| Patients' total score | 0 | 24 | 7.5 | 5.1 | 0 | 28 | 5.9* | 5.6 |
| Physicians' assessment | | | | | | | | |
| General assessment | 0 | 4 | 2.2 | 0.9 | 0 | 4 | 1.8 | 1.0 |
| Scalp involvement | 0 | 4 | 1.1 | 1.3 | 0 | 4 | 0.9 | 1.2 |
| Face involvement | 0 | 4 | 0.4 | 0.8 | 0 | 4 | 0.4 | 0.8 |
| Frontal trunk involvement | 0 | 4 | 0.7 | 1.1 | 0 | 4 | 0.6 | 1.1 |
| Back involvement | 0 | 4 | 0.8 | 1.1 | 0 | 4 | 0.7 | 1.1 |
| Upper limbs involvement | 0 | 4 | 1.6 | 1.0 | 0 | 4 | 1.3 | 1.0 |
| Palms involvement | 0 | 3 | 0.2 | 0.6 | 0 | 3 | 0.2 | 0.6 |
| Lower limbs involvement | 0 | 4 | 1.4 | 1.2 | 0 | 4 | 1.1 | 1.1 |
| Soles involvement | 0 | 3 | 0.2 | 0.7 | 0 | 3 | 0.2 | 0.6 |
| Physicians' total score | 2 | 33 | 8.6 | 5.7 | 0 | 33 | 7.0** | 5.9 |
| PASI | 1 | 64 | 9.8 | 9.5 | 0 | 61 | 8.0*** | 9.6 |
| BPSS | 2 | 57 | 16.1 | 9.8 | 0 | 57 | 12.8† | 10.6 |

* $p = 0.018$.

** $p = 0.026$.

*** $p = 0.090$.

† $p = 0.010$.

Complementary medicine includes therapeutic modalities that derive from traditional and philosophical systems of medicine, which view health and disease in the context of human totality of body and mind. These practices are perceived as outside of the mainstream domain of medicine[5,6,7,8,9,10,11,12,13,14,15,16,17,18].

QoolSkin is an herbal topical medication indicated for the treatment of patients with psoriasis. QoolSkin ingredients include riboflavin, citrus medica limonum (lemon) juice, vinegar, taraxacum oficinalis (danadelion) extract, cereus grandiflorus (cactus) flower extract and opuntia coccinellifera fruit extract, ascorbic acid, calcium ascorbate, sodium ascorbate, and tartaric acid.

**TABLE 2**
**Assessment of Psoriasis Severity Before and After QoolSkin Treatment, Stratified by Gender**

| | Before QoolSkin Treatment | | | | After QoolSkin Treatment | | | |
|---|---|---|---|---|---|---|---|---|
| | Min. | Max. | Mean | SD | Min. | Max. | Mean | SD |
| Men | | | | | | | | |
| Patients' total score | 1.0 | 24.0 | 7.9 | 5.4 | 0.0 | 28.0 | 7.2 | 6.4 |
| Physicians' total score | 2.0 | 33.0 | 9.2 | 6.0 | 0.0 | 33.0 | 8.0 | 6.2 |
| PASI | 1.8 | 63.6 | 11.4 | 10.7 | 0.0 | 61.2 | 10.4 | 10.8 |
| BPSS | 3.0 | 57.0 | 17.0 | 10.6 | 0.0 | 57.0 | 15.2 | 11.8 |
| Women | | | | | | | | |
| Patients' total score | 0.0 | 22.0 | 7.0 | 4.8 | 0.0 | 15.0 | 4.4 | 4.2 |
| Physicians' total score | 2.0 | 23.0 | 8.0 | 5.4 | 0.0 | 23.0 | 5.9 | 5.3 |
| PASI | 0.9 | 46.2 | 8.0 | 7.6 | 0.0 | 46.2 | 5.4 | 7.2 |
| BPSS | 2.0 | 35.0 | 15.0 | 8.8 | 0.0 | 34.0 | 10.3 | 8.5 |

**TABLE 3**
**Assessment of Psoriasis Severity According to the Length of QoolSkin Treatment**

| | Length of Treatment | N | Mean | Minimum | Maximum |
|---|---|---|---|---|---|
| BPSS reduction | <30 days | 16 | 0.4% | –35.7% | 58.8% |
| | 30–60 days | 15 | 9.7% | –80.0% | 100.0% |
| | 60–90 days | 12 | 9.3% | –75.0% | 81.3% |
| | 90–120 days | 30 | 26.3% | –55.6% | 100.0% |
| | >120 days | 25 | 51.4% | –11.1% | 100.0% |
| PASI reduction | <30 days | 16 | 3.3% | –9.7% | 40.0% |
| | 30–60 days | 15 | 15.7% | –7.8% | 100.0% |
| | 60–90 days | 13 | 16.3% | –82.8% | 100.0% |
| | 90–120 days | 30 | 23.1% | –92.9% | 100.0% |
| | >120 days | 25 | 48.4% | –9.4% | 100.0% |

We observed that the effect of QoolSkin was pronounced after longer periods of application. It is possible that it takes a long time for the herbal ingredients of QoolSkin to effect the skin manifestations of psoriasis, due to the delayed effect on the mind of the patients.

The response to QoolSkin treatment varied according to the treating investigator. As treatment by complementary medicine involves not only the pharmaceutical effects of the medication, but also the attitudes and feelings of the patients, it is possible that the superior effect of QoolSkin observed by one of the investigators (AS) is related to a different approach to complementary medicine of this investigator. Another possible explanation is that the pronounced effect observed in patients treated by AS is related to the fact that these patients applied QoolSkin three times per day and for a longer period of time.

It is possible that the outcomes of QoolSkin treatment observed in the study were attributed to placebo effect or to the lubrication of the QoolSkin formula. However, these preliminary results are encouraging, as the positive effects of herbal treatment were observed concomitantly in four separate research sites. We suggest that a double-blind, placebo-controlled study should be conducted to further assess the efficacy of QoolSkin in patients with psoriasis.

**TABLE 4**
**Assessment of Psoriasis Severity According to the Treating Investigator**

| | Study Site | Investigator | N | Mean | Minimum | Maximum |
|---|---|---|---|---|---|---|
| BPSS reduction* | 1 | RS | 14 | 14.9% | –14.3% | 100.0% |
| | 2 | ADC | 19 | 3.9% | –80.0% | 81.3% |
| | 3 | RY | 15 | 11.0% | –55.6% | 82.1% |
| | 4 | AS | 48 | 37.8% | –35.7% | 100.0% |
| | | Total | 96 | 23.6% | –80.0% | 100.0% |
| PASI reduction | 1 | RS | 14 | 27.1% | 0.0% | 100.0% |
| | 2 | ADC | 20 | 12.3% | –92.9% | 100.0% |
| | 3 | RY | 15 | 11.6% | –75.0% | 97.1% |
| | 4 | AS | 48 | 32.0% | –85.7% | 100.0% |
| | | Total | 97 | 24.1% | –92.9% | 100.0% |

* $p = 0.002$.

**TABLE 5**
**Assessment of Psoriasis Severity Before and After QoolSkin Treatment in 48 Patients Treated by AS**

| | Before Treatment | | | | After Treatment | | | |
|---|---|---|---|---|---|---|---|---|
| | Min. | Max. | Mean | SD | Min. | Max. | Mean | SD |
| Patients’ assessment | | | | | | | | |
| General assessment | 1.0 | 5.0 | 2.6 | 1.3 | 0.0 | 5.0 | 1.5 | 1.1 |
| Pruritus | 0.0 | 5.0 | 2.0 | 1.5 | 0.0 | 4.0 | 0.9 | 1.2 |
| Face involvement | 0.0 | 2.0 | 0.1 | 0.4 | 0.0 | 2.0 | 0.1 | 0.3 |
| Nails involvement | 0.0 | 5.0 | 0.3 | 0.9 | 0.0 | 5.0 | 0.3 | 0.9 |
| Palms involvement | 0.0 | 0.0 | 0.0 | 0.0 | 0.0 | 0.0 | 0.0 | 0.0 |
| Soles involvement | 0.0 | 2.0 | 0.0 | 0.3 | 0.0 | 2.0 | 0.0 | 0.3 |
| Genital involvement | 0.0 | 3.0 | 0.2 | 0.6 | 0.0 | 3.0 | 0.2 | 0.6 |
| Patient total score | 1.0 | 13.0 | 5.2 | 3.1 | 0.0 | 11.0 | 3.0 | 2.7 |
| Physicians' assessment | | | | | | | | |
| General assessment | 0.0 | 4.0 | 2.1 | 0.9 | 0.0 | 4.0 | 1.4 | 1.0 |
| Scalp involvement | 0.0 | 4.0 | 0.9 | 1.3 | 0.0 | 4.0 | 0.7 | 1.1 |
| Face involvement | 0.0 | 2.0 | 0.1 | 0.4 | 0.0 | 2.0 | 0.1 | 0.4 |
| Frontal trunk involvement | 0.0 | 3.0 | 0.4 | 0.9 | 0.0 | 3.0 | 0.3 | 0.7 |
| Back involvement | 0.0 | 3.0 | 0.5 | 0.9 | 0.0 | 3.0 | 0.4 | 0.7 |
| Upper limbs involvement | 0.0 | 3.0 | 1.6 | 0.9 | 0.0 | 3.0 | 1.1 | 0.9 |
| Palms involvement | 0.0 | 1.0 | 0.0 | 0.1 | 0.0 | 1.0 | 0.0 | 0.1 |
| Lower limbs involvement | 0.0 | 4.0 | 1.4 | 1.1 | 0.0 | 3.0 | 1.0 | 0.9 |
| Soles involvement | 0.0 | 1.0 | 0.0 | 0.1 | 0.0 | 1.0 | 0.0 | 0.1 |
| Doctor total score | 2.0 | 21.0 | 7.1 | 4.3 | 0.0 | 21.0 | 5.0 | 4.3 |

### DECLARATION

The authors have served as investigators for BMZ as well as other companies that manufacture treatments for psoriasis.
